# Optimization of Cement-Based Mortar Containing Oily Sludge Ash by Response Surface Methodology

**DOI:** 10.3390/ma14216308

**Published:** 2021-10-22

**Authors:** Mubarak Usman Kankia, Lavania Baloo, Nasiru Danlami, Wan Nurliyana Samahani, Bashar S. Mohammed, Sani Haruna, Ahmad Hussaini Jagaba, Mahmud Abubakar, Effa Affiana Ishak, Khalid Sayed, Noor Amila Bt Wan Zawawi

**Affiliations:** 1 Civil and Environmental Engineering Department, Universiti Teknologi PETRONAS, Seri Iskandar 32610, Malaysia; mubarak_18001828@utp.edu.my (M.U.K.); wan.nurliyana_22512@utp.edu.my (W.N.S.); bashar.mohammed@utp.edu.my (B.S.M.); sani_17000823@utp.edu.my (S.H.); ahmad_19001511@utp.edu.my (A.H.J.); effa_18001790@utp.edu.my (E.A.I.); khalid_19000239@utp.edu.my (K.S.); amilawa@utp.edu.my (N.A.B.W.Z.); 2 Civil Engineering Department, Bayero University, Kano PMB 3011, Nigeria; nassday@live.com; 3 Civil Engineering Department, Federal University of Technology, Minna PMB 65, Nigeria; mahmud1879@futminna.edu.ng

**Keywords:** oily sludge, cement, compressive strength, petroleum hydrocarbon

## Abstract

In the industries of petroleum extraction, a large volume of oily sludge is being generated. This waste is usually considered difficult to dispose of, causing environmental and economic issues. This study presented the novel experimental method of manufacturing mortar used in civil construction by cement and oily sludge ash (OSA). The defined method was described with a logical experimental study conducted to examine a feasible manufacturing method for casting cement-based mortars by partially replacing cement with OSA. Replacement concentrations for OSA ranged from 0 to 20 percent by cement weight, while the water-to-cement (w/c) ratio was varied from 0.4 to 0.8, and the amount of sand was kept constant. The strengths and absorption rate of the mortar were monitored for 28 days. The OSA contains a crystalline structure with packs of angular grains. Because of OSA in the cement-based mortar mixtures and water-to-cement ratios, the mechanical strength was improved significantly. However, the water absorption trend increased linearly. Using variance analysis, the influence of OSA and w/c ratio on the behavior of mortar was acquired. The developed models were significant for all *p*-value reactions of <5%. Numerical optimization results showed that the best mixture can be obtained by replacing 8.19 percent cement with OSA and 0.52 as a ratio of w/c.

## 1. Introduction

The management of waste has drawn the concern of industrialists and researchers in recent years to decrease environmental hazards and problems. The globe is observing a constant growth in the generation of waste products from the evolution of industrial projects consumed by man. Numerous efforts have been made to invent and use scientific and economic approaches to maximize the advantages of these wastes [1]. Despite the works and studies, several gases and solids drain into the environment without any treatment, leading to severe risks for water and air. The issue is how the wastes can be utilized to avoid environmental damage that is reflected in climate change.

The remains of the petroleum industry are one of the most crucial industrial by-products in the world, especially in countries that are exporting oil such as Malaysia. In Malaysia, one typical refinery with the capacity to manufacture 105,000 barrels/day can produce another 50 tons of oily sludge (petroleum sludge) per year. The tremendous amount of wastes generated is alarmingly increasing as the production of crude oil becomes more important in time because crude oil is the sole source of energy and acts as feedstock in refineries [2]. Oily sludge is the result of several processes of the oil industry (wells drilling, crude oil pools, refining, transporting, and storage tanks) [3]. Oily sludge is a hazardous waste containing a complex mixture of different emulsified petroleum hydrocarbons, water, heavy metals, and solid particles. Oily sludge contains large amounts of crude oil, heavy metal ions, inorganic salts, polycyclic aromatic hydrocarbons (PAHs), and other toxic substances that contaminate the soil, surface, and groundwater [4,5]. According to the Resources Conservation and Recovery Act (RCRA), oily sludge is a hazardous waste that affects the environment and the living things if it is disposed of improperly [6]. Effective management of the oily sludge waste is urgently required, where its resulting reuse and recycling represent ideal opportunities for sustainable development [7].

There are different techniques for the treatment of oily sludge, and the choice depends on the desired aim to be achieved [8]. Various oily sludge treatment methods include centrifugation, froth flotation, solidification/stabilization, solvent extraction, surfactant EOR (enhanced oil recovery), landfarming, pyrolysis, biodegradation, photocatalysis, ultrasonic treatment, and incineration [9,10,11,12,13]. Researchers have concluded that it is ineffective to implement a typical method to fully remove the dangers of the oily sludge without any side-effects, and these methods may either partially or fully decrease the harmful substances in petroleum sludge to a minimum acceptable level [14].

Thermal incineration is among the available techniques for the treatment of oily sludge [15]. The thermal method helps to remove grease and oil that could retard the setting time and the development of strength, which affect the encapsulation of heavy metals in the solidified matrix. The method can also decrease all significant PAHs, but it is associated with a high temperature of operation [16]. Besides, the generated solid residue (oily sludge ash, OSA) from the thermal technique poses some issues, challenges, and problems concerning environmental safety and public health protection. The encapsulation of heavy metals is one of the major concerns that decrease their migration into the environment. The stabilization and solidification (SS) method is a successful and efficient process of disposal commonly employed to deactivate or decrease the harmful effects of heavy metals’ migration into the environment [17]. SS is a quick and cheap treatment strategy of OSA, fulfilling the need to immobilize hazardous wastes by converting them into a less harmful or dissolvable matrix (i.e., stabilization) and confining the deactivated wastes into a durable matrix form, i.e., solidification [4]. The SS technique involves the use of cement as a binder to seal or encapsulate the hazardous waste in a closed and inactive space through the physicochemical process. The SS method offers a good performance of mechanical properties, and pozzolanic materials are commonly added to increase the SS performances.

In construction industries, mortar is commonly used as a construction material, and the ingredients include water, cement, and fine aggregates. These three basic components are proportioned appropriately and mixed homogeneously to produce mortar with the aid of an admixture to enhance the chosen performance of specific physical and mechanical characteristics such as density, hardness, corrosion resistance, durability, compressive strength, and flexural strength of mortar [18]. These properties mainly depend upon the appropriate selection of water/cement ratio, properties of the cement, and fine aggregate. These ingredients offer significant benefits either to replace them partially or fully to produce mortar with the desired properties. Numerous waste materials such as agricultural by-products and industrial wastes that contain amorphous silica have been used as partial replacement materials of cement in the construction industries, and they are known as cement replacement materials (CRM). Cement has been replaced with, in some cases, fly ash, silica fume, slag, coconut shell ash, stainless steel waste, and palm kernel shell ash [19,20,21]. Similarly, the fine aggregate was substituted with the waste materials to produce lightweight mortar/concrete [22]. Studies revealed that up to 40% of cement was replaced and showed a significant enhancement of compressive strength compared to the control made with 100% cement [16].

Pinheiro and Holanda [23] partially substituted kaolin material with oily sludge up to 5 wt.% in the manufacture of porcelain stoneware tile. It was revealed that the optimum replacement of kaolin is 2.5 wt.% of oily sludge, producing the porcelain stoneware tiles with safe leaching limits. Moreover, the results indicated that incorporating more oily sludge into tile preparations led to a declination in apparent density, linear shrinkage, and flexural strength, while the water absorption increased. In addition, Sengupta et al. [24] used oily sludge in manufacturing acceptable masonry bricks. The oily sludge addition in the mixture reduced the needed water and fuel in the manufacturing process. The cured bricks achieved all the Indian Standard requirements. Souza et al. [25] added oily sludge in the manufacture of clay-based ceramics (roofing tiles and hollow bricks) up to 30 wt.% of waste as a substitute for natural clay, and the result revealed that the heavy metals were within allowable leaching limits. Usman et al. [5] incorporated OSA in fly ash-based geopolymer mortar and found that the 28 day compressive strength for the mixture made with 10 wt.% OSA was highest (31 MPa).

The techniques for the efficient reuse of oily sludge and effective and safe oily sludge disposal are very important to reduce the problems associated with oily sludge. The present study aimed to thermally treat petroleum sludge to remove oil and grease that affect the hydration reaction of cement and strength development and reduce the level of PAHs and heavy metals in the oily sludge. The response surface methodology was employed to optimize the mortar proportions where the amount of OSA and the ratio of water to cement were used as independent variables. Furthermore, the performance of the OSA in the mortar mixture was assessed through compressive strength, flexural strength, and water absorption.

## 2. Materials and Methods

### 2.1. Materials

The oily sludge was obtained from a local refinery in Malaysia. It was found to be viscous, black, and semi-solid. The Department of Environment, Malaysia (2010 guidelines) categorized oily sludge as a scheduled waste (SW314). For the production of the cement-based mortar, locally available natural-siliceous sand having a fineness modulus of 2.52 and an average particle size of 400 μm, agreeing with ASTM C33/C33M-13 [26], was used. Castle brand type I of ordinary Portland cement, conforming to the ASTM C150 specifications and having a specific gravity of 3.15 and its chemical composition determined by XRF analysis, presented in Table 5, was used in the mortar production.

### 2.2. Experimental Methods

#### 2.2.1. Characterization of Oily Sludge

##### Moisture Content and Total Solid Residue

**Figure 1:**
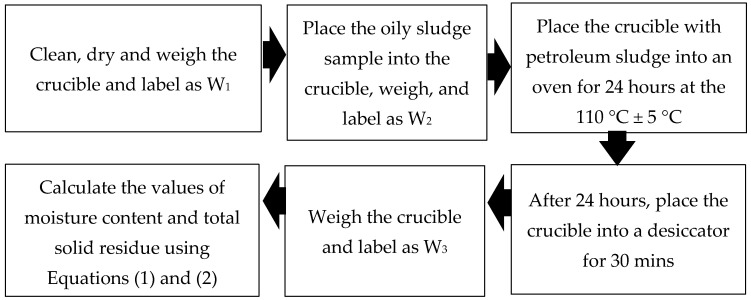
Process flow for the determination of moisture content and total solid residue.

The moisture content of oily sludge was measured using the oven-drying method, following the ASTM D-3173 standard guidelines. Three crucibles were cleaned, dried, and weighed and the values were labeled as W_1._ The oily sludge was poured into crucibles and weighed, and the values obtained were designated as W_2._ The crucibles were placed in an oven and dried at a temperature of 110 °C ± 5 °C. After 24 h, the crucibles were removed from the oven and cooled in a desiccator; the crucibles were weighed again and denoted as W_3_. The moisture content and total solid residue (TSR) were calculated using Equations (1) and (2), respectively. The process has been illustrated using a flow process shown in Figure 1.

(1) $$\mathrm{Moisture}\;\mathrm{Content}=\frac{\left(W_{2}-{\;W}_{1}\right)-\left(W_{3}-{\;W}_{1}\right)}{\left(W_{2}-{\;W}_{1}\right)}\times 100\%$$

(2) $$\mathrm{Total}\;\mathrm{Solid}\;\mathrm{Residue},\;\left(\mathrm{TSR}\right)=\frac{\left(W_{3}-{\;W}_{1}\right)}{\left(W_{2}-{\;W}_{1}\right)}\times 100\%$$

where W_1_ = weight of crucible, W_2_ = weight of crucible with oily sludge before oven drying, W_3_ = weight of crucible with oily sludge after oven drying.

##### Petroleum Hydrocarbons Analytical Procedure

**Figure 2:**
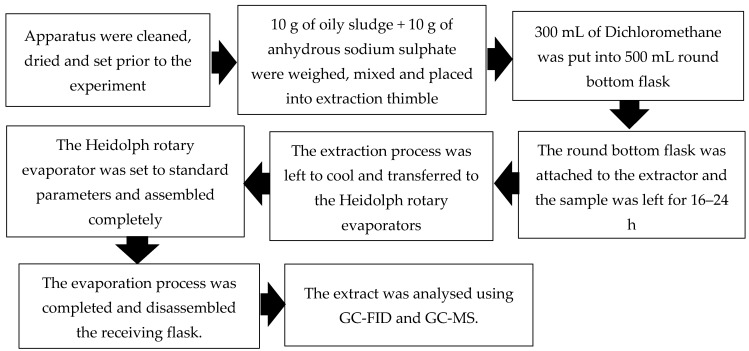
Process flowchart for the determination of petroleum hydrocarbons.

Before the determination of petroleum hydrocarbons (PHCs) using a gas chromatograph–mass spectrometer (GC–MS) and gas chromatograph–flame ionization detector (GC-FID) by Carl Zeiss AG, Jena Germany, an oily sludge sample was prepared through the Soxhlet extraction technique for easy detection of PHCs in the oily sludge [27]. A 10 g sample of oily sludge was added with 10 g of anhydrous sodium sulfate placed in the extraction apparatus. Then, 300 mL of dichloromethane (DCM) was placed into a 500 mL round-bottom flask. DCM was used as an organic solvent as the targeted hydrocarbon compounds such as PAHs have limited solubility. The round-bottom flask was attached to the extractor and left for 16 to 24 h. The extraction process was left to cool before it was transferred to the Heidolph rotary evaporator, ProfiLab24, Landsberger, Berlin, which was set to the standard parameters. As the evaporation process was completed, the receiving flask was disassembled, and the extract was collected in vials before conducting the GC-MS test. A stock solution of a PAH mixture standard (Restek Corp, Bellefonte, PA, USA) was used to prepare standard solutions of 17 concentrations from 0.001 to 9.0 ppm for calibration. PAH in the oily sludge specimens was examined using a 6890N GC and 5973N MS with 7683 auto-injectors (Agilent Technologies Inc., Santa Clara, CA, USA). The process flowchart is shown in Figure 2.

##### Total Organic Carbon

Total organic carbon (TOC) analysis was conducted to determine the amount of carbon present in the oily sludge sample. It was used as an indicator of the degradation of the oily sludge sample. The proposed method for this characterization was the combustion catalytic oxidation method using the TOC analyzer machine by Shimadzu, North America. Before the analysis, the oily sludge sample was prepared in the form of an allowable solid. For the sample preparation, the oily sludge was dried and ground into powder form before mixing with 10% hydrochloric acid (HCl). The mixture was then dried in an oven at a temperature of 105 °C for 3 h. The sample was heated at 680 °C in an oxygen-rich environment inside total carbon (TC) combustion tubes filled with a platinum catalyst. Through the oxidation, carbon dioxide was generated and detected using an infrared gas analyzer (NDIR). By comparing the calibration curve formula, the concentration of TC was determined. After obtaining the TC and inorganic carbon (IC), they were substituted in Equation (3) to obtain TOC.

(3) TOC=TC− IC

##### pH Analysis

The pH value was to ensure that the acidity of the oily sludge remains within the permissible limit. Here, 30 g of the oven-dried sample, which was used for the moisture content test, was reused and diluted into a 40 mL hexane solution before measuring the pH value, and the OHAUS ST3100-F benchtop pH meter (Ohaus Corporation, Parsippany, NJ, USA) was used as the equipment. Hexane was chosen because it is an organic solvent and would not affect the pH of the oily sludge sample [28].

#### 2.2.2. Preparation and Characterization of Oily Sludge Ash

Air suction filters were used to remove the water from the oily sludge and then placed into a laboratory oven at 105 °C to evaporate the water that remained in the oily sludge for 6 h. About 500 g of the dried oily sludge was blended with additives of 1 mole of calcium hydroxide (Ca (OH)_2_) and 1 mole of sodium bicarbonate (NaHCO_3_). The additives aided in the degradation of the polycyclic aromatic hydrocarbons [16]. The oily sludge was burnt in the laboratory muffle furnace for approximately 2 h at a temperature of 600 °C (most metals volatilize at 650 °C) [29]. Oily sludge ash (OSA) appeared to have larger particles and was ground in a laboratory milling machine and passed through a 45 µm sieve [30]. The chemical composition of OSA was determined by qualitative and quantitative measurement and analysis using X-ray fluorescence (XRF) spectroscopy analysis, and 15 g of the OSA sample was used for the test. Field-emission scanning electron microscopy was used to analyze the OSA sample to obtain the surface morphology. X-ray diffraction (XRD) analysis of the OSA was conducted to obtain the crystalline patterns and compositions of minerals. The sample was scanned at room temperature from 0 to 80° 2θ using a 0.0262 scan step size with Cu Kα radiation. The XRD pattern intensities were recorded using a constant divergence slit of 0.38 mm along with Cu as the anode material by using two reflection coefficients, Kα1 of 1.5406 and Kα2 of 1.5444. The mineral identification was carried out with HighScore Plus software (version 5.1, Malvern Panalytical Ltd., Malvern, United Kingdom).

#### 2.2.3. Response Surface Methodology

The response surface methodology (RSM) aims to optimize the efficiency of dependent variables through changing input variables [5]. The RSM involves experiment, modeling, and data analysis. In this study, the RSM was used to examine the influence of the OSA and water–cement ratio. Numerical optimization was performed to study the most suitable mix formulation by enhancing the strengths and limiting water absorption. Design expert software was used for experimental design. Using the rotatable (k < 6) option of the central composite design (CCD), two input variables of mix formulations of the mortar were carefully chosen. The OSA ranging from 0 to 20 percent by weight with a water–cement ratio of 0.4 to 0.8 is shown in Table 1. Table 2 also shows the mixed formulations of the cement-based mortar according to the RSM design. The experimental scheme consisted of two independent variables, five levels, and 10 experimental runs. The outputs of this study included compressive strength, flexural strength, and water absorption. The software established ten mix combinations for each response with two randomized duplications. The software used two duplicates of the central points to develop the accuracy of experiments against any possible error.

#### 2.2.4. Mix Formulation, Casting, Curing, and Testing of Mortar Samples

The mixed proportions of the cement-based mortar used in the present study are shown in Table 3. OSA, OPC, and siliceous sand were the main materials for the mortar mixes. The cement-to-sand ratio of 1:3 and water-to-cement ratios of 0.4 to 0.8 were adopted in this study. The quantities of cement, fine aggregates, and OSA were measured and then thoroughly mixed in a small Hobart mixer for about 2 min. Furthermore, the required amount of water was gently added into the rotating mixer and then mixed for 3 to 5 min until it became consistent and homogeneous. Each mix formulation was then cast into the six molds of 50 mm cube dimensions and three prisms of 500 mm × 100 mm × 100 mm prism dimensions and compacted with the help of a table vibrator. After 24 h, the hardened specimens of the mortar were removed from the molds and placed into a water bath for curing at laboratory temperature. Before the compressive strength test, the cubes were removed from the curing tank and a towel was used to clean the water on the surface on the samples. A digital UCS strength testing machine of 3000 kN capacity was used to determine the UCS strength of the geopolymer mortar specimens according to the standard test method (ASTM C109/C109 M standard) [5] at the age of 28 days. Prismatic beams (100mm × 100mm × 500 mm) were used to assess the flexural strength of the beams subjected to a three-point bending (TPB) test. The test was conducted according to the criteria described in ASTM C293M-10 [31]. Equation (4) was used to quantify the flexural strength of the samples subjected to the TPB test.

(4) $$\mathrm{Flexural}\;\mathrm{strength}=\frac{3PL}{2bd^{2}}$$

where *P* = failure load, *L* = effective span of the beam, and *B* and *h* = the width and height of the beam, respectively.

A water absorption test was performed following ASTM C642-13 [32]. For the water absorption test, the specimens were dried in an oven at 105 ± 5 °C for 24 h and placed in a desiccator to cool. Immediately upon cooling, the specimens were weighed until constant mass (*Wd*). Then, they were immersed in water for 24 h and the saturated weight was taken as *Ws*. The water absorption was calculated by the following formula, Equation (5).

(5) $$\mathrm{Water}\;\mathrm{absorption}=\frac{Ws-W_{d}}{W_{d}}\times 100$$

## 3. Results and Discussion

### 3.1. Characterization of Oily Sludge

The characteristic studies of raw oily sludge are shown in Table 4. The calculated moisture content for the oily sludge sample is 61.4%, while the total solid residue contained in the sample is 35.8%. The oily sludge has a pH value of 6.44, which is quite acidic but almost neutral. The typical pH range was proven by other researchers to range from 6.0 to 7.0 [33]. The acidic behavior of the sludge may create favorable room for the growth of microorganisms in the soil where it was disposed of [28]. Hence, the pH value of oily sludge had proven that the oily sludge is acidic and requires proper preparation before disposal. Total organic carbon was achieved using a TOC analyzer aiming to quantify the total petroleum hydrocarbon weight fractions. The concentration of TOC was 2.2%, giving a brief measure of organic carbon in any form such as petroleum hydrocarbons and natural matter [34]. Through GC-FID, total petroleum hydrocarbon (TPH) can be divided into four organic ranges—diesel range organics (DRO), gasoline range organics (GRO), oil range organics (ORO), and residual range organic (RRO).

**Figure 3:**
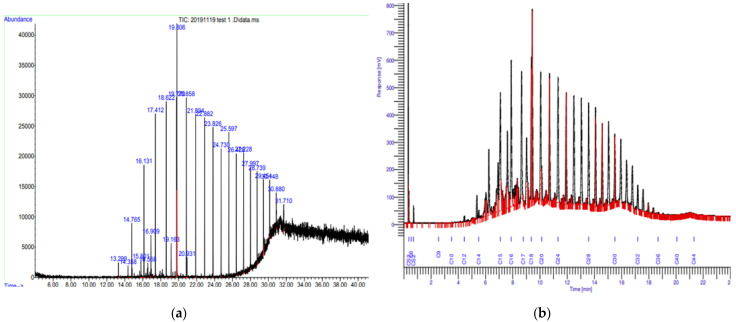
(**a**) GC-MS, and (**b**) GC-FID chromatographs of oily sludge sample.

Through GC-MS analysis, the type of compounds present in the sample was detected using the retention time reading, while the peak areas were used to calculate the amount of the compounds present in the oily sludge sample. From Figure 3, notably, 27 peak areas were detected and 86.88% of the compounds found were classified as hydrocarbons, which are also known as the first family of organic compound class [35]. Two major hydrocarbon compounds in the oily sludge consist of 0.62% PAH, and the remainder are classified as saturated aliphatic compounds [36].

The GC-FID chromatographs above show that the carbon range present in the oily sludge sample ranged from C_12_ to C_44_. Moreover, 84.3% DRO was detected, typically ranging from C_12_ to C_28_. Almost none of the GRO were detected. Hence, the analysis had proven that oily sludge was highly contaminated by diesel fuel. In addition, the remaining carbons that existed in the sample were categorized as ORO and RRO. Diesel fuel oil is a complex mixture containing approximately 80–90% aliphatic hydrocarbons and 10–20% aromatic hydrocarbons.

### 3.2. Characterization of Oily Sludge Ash

**Figure 4:**
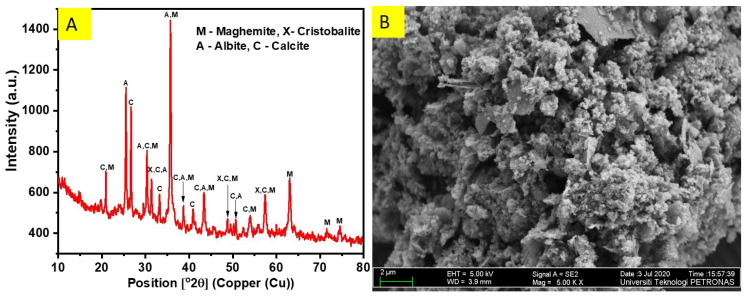
Oily sludge ash (**A**) XRD pattern and (**B**) FESEM image.

**Figure 5:**
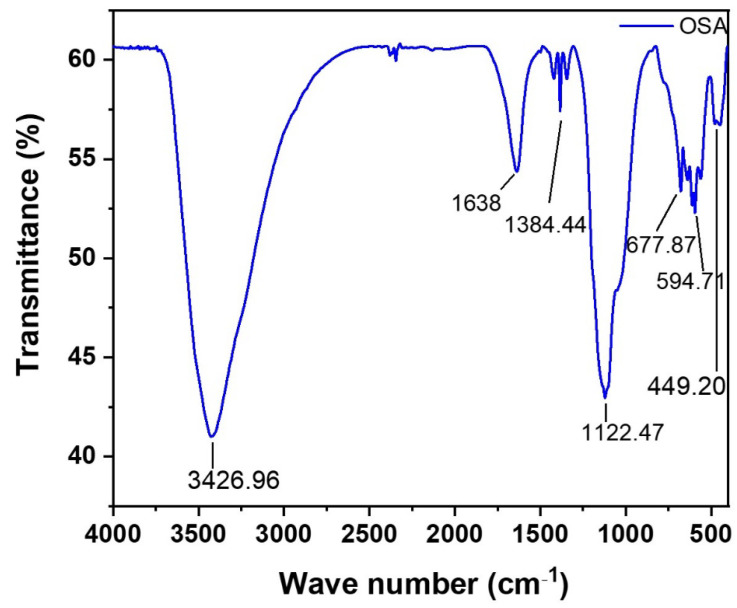
Oily sludge ash FTIR.

The XRD pattern and micrograph of the OSA are shown in Figure 4. From Figure 4A, the XRD result of the OSA sample shows that the OSA contained mineral phases comprising albite, calcite, cristobalite, and maghemite. The morphology of OSA was obtained using the FESEM and is shown in Figure 4B as displaying packs of angular grains. The chemical compositions of ordinary Portland cement (OPC) and OSA were determined using X-ray fluorescence (XRF) and are displayed in Table 5. The sum of SiO_2_, Al_2_O_3_, and Fe_2_O_3_ for the OSA was 70.60% along with CaO of 9.82%; thus, it conforms to the ASTM C618-15 specification [37]. Therefore, based on the crystalline structure, morphology, and chemical composition of OSA, it can be used as a potential cement replacement material for mortar/concrete. Figure 5 illustrates the pattern of FTIR for OSA. The OSA showed broad absorbance peaks. The 3426.96 cm^−1^ band was due to hydroxyl groups (O – H stretching); the 1638 cm^−1^ band was attributed to C = O stretching of carbonyl/carboxylic groups; the 1420.57 cm^−1^ band was attributed to methyl (–CH_3_) and methylene (–CH_2_) groups; the 1384.44 cm^−1^ band was associated with the methyl group –CH_3_; the 1122.47 cm^−1^ was linked to C–O–C; the 594 cm^−1^ aromatic bands were attributed to aromatic carbon–carbon rocking vibrations. OSA has hydrophobic groups, including methylene and methyl aromatic rings.

### 3.3. Compressive Strength

**Figure 6:**
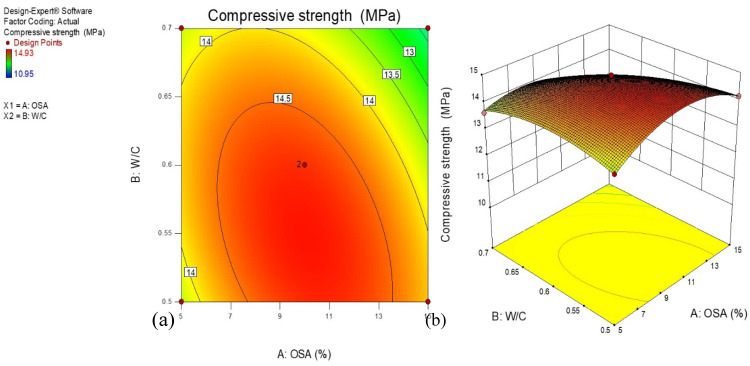
(**a**) 2-D and (**b**) 3-D plots for compressive strength development of cement-based mortar.

Figure 6 illustrates the unconfined compressive strength results of the cement-based mortar incorporating oily sludge ash at different water-to-cement ratios at 28 days. Furthermore, the plot of the contours for the developed compressive strength model is graphically displayed through a two-dimensional plot, as depicted in Figure 6a. It is worth mentioning that all the contour lines elliptically demonstrate a perfect interaction between input variables, the amount of OSA, and the water–cement ratio. The reddish portion on the contour plot designates an excellent mixture that yields the best strength values. Figure 6b, a three-dimensional surface diagram, reveals the influence of OSA inclusion in the cement-based mortar mixtures, and water-to-cement ratios improved the compressive strength significantly. For any W/C ratio, increasing the amount of OSA in the mortar from 0 to 20 wt.% increased the compressive strength of the mortar considerably to a certain limit. This effect was extremely pronounced in the mix with a W/C ratio of 0.50. With the further increase in the W/C ratio and amount of OSA, the compressive strength decreased noticeably. The strength decrease is attributed to the decrease in reactions, resulting in gel formation.

### 3.4. Flexural Strength

**Figure 7:**
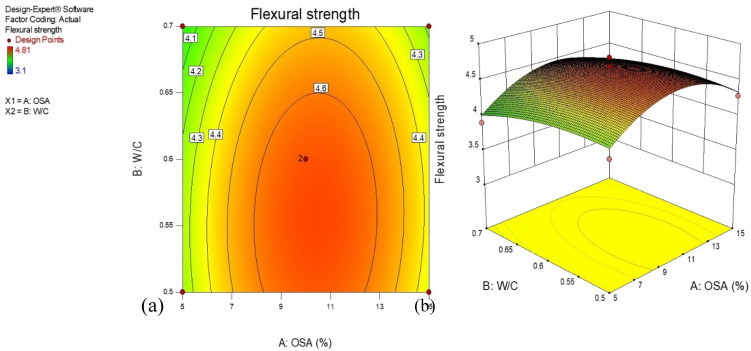
(**a**) 2-D and (**b**) 3-D plots for flexural strength of cement-based mortar.

Figure 7 displays the flexural strength of cement-based mortar containing OSA at 28 days. In addition, the flexural strengths of the samples were increased when the OSA was added to the mixtures. Similarly, the ratios of water to cement had a considerable influence on the flexural strength growth. Figure 7a presents a 2-D surface graph of the variations in the flexural strength response model for the input variables considered. It is seen that the whole contour lines were linear in shape, displaying the good interactions between the amount of OSA and W/C ratios. The model was found to have a good correlation between the independent variables. It can be observed that the OSA contributed significantly toward the improvement of the flexural strength of the geopolymer mortar. It is interesting to note that the flexural strength of the cement-based mortar increased with the amount of OSA for a given W/C ratio to a certain level. The minimum flexural strength was obtained in the greenish portions, while the optimum was at the reddish area of the contour graph. Figure 7b displays a 3-D graph of the flexural strength illustrating all the independent factors and their significant effect on the mortar flexural strength. The flexural strength of the cement mortar containing OSA was improved when the two variables were increased to a certain level. However, the flexural strength reduced when the weight percentage of OSA and the ratio of water to cement increased beyond the optimum values.

### 3.5. Water Absorption

**Figure 8:**
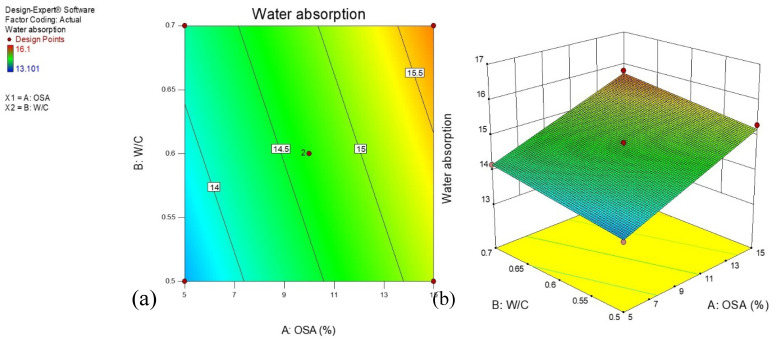
(**a**) 2-D and (**b**) 3-D plots for flexural strength of geopolymer mortar.

A water absorption test was conducted to evaluate the effect of OSA and w/c ratio on a cement-based mortar. Figure 8a displays the 2-D contour plots for the water absorption model of mortar containing OSA. The contour lines were linear in shape, illustrating an ideal interaction between the amount of OSA and the ratio of water to cement. The established model revealed an ideal synergy among the input variables. The bluish portions on the contour plot showed the best combination that produced minimum values of water absorption, while the reddish areas indicated the region of the maximum water absorption of the optimized cement-based mortar. Figure 8b illustrates the 3-D response surface graph, which explains the influence of the input variables on the water absorption of the mortar. It is seen from the graph that the two input variables affected the water absorption significantly. It is observed that the water absorption trend increased linearly with the increase in both the ratio of water to cement and the amount of OSA in the mortar. This could be associated with the evaporation of the excess water in the mortar matrices and leaving behind pores that result in decreasing the dense and compact mortar matrices, allowing water infiltration.

### 3.6. Statistical Interpretation of the Test Results

Table 6 presents the analysis of variance for response models. All the established models were analyzed statistically and validated. The analysis was carried out at a 5% level of significance to evaluate the significance of the input variables of the experiment. Compressive and flexural strength along with water absorption for the cement-based mortars were selected as the independent variables in the present study, while the amount of PSA and the ratio of water to cement were carefully selected as independent variables. The terms of the models’ F-values of 155.03, 34.21, and 302.12 imply that the models of the mortar were significant, having *p*-values of <0.05. For compressive strength, flexural strength, and water absorption, there was only a 0.01, 0.22, and 0.01% chance, respectively, that an F-value this large could occur due to noise. Values of Prob > F less than 0.0500 indicate that the model terms for compressive strength are significant. In this case, A, B, AB, A^2^, and B^2^ were significant model terms. Similarly, A^2^ and B^2^ were significant model terms for flexural strength, while water absorption had A and B as significant model terms. Values greater than 0.1000 indicate that the model terms are not significant. The fitness and quality of the established model might be described by its high level of correlation. The quality of the models could be measured using a lack of fit; lower lack-of-fit values indicate better models. As revealed in Table 6, the lack-of-fit p-values for all the models were greater than 0.05, indicating that the lack of fit was not significant and consequently designated that the response models were fit outstandingly.

The models’ quality might also be studied through the R-squared value. As shown in Table 7, the high R-squared values of 0.9949, 0.9771, and 0.9885 for the compressive strength, flexural strength, and water absorption model, respectively, show a good measure of correspondence between the experimental and predicted results. It is also worth mentioning that the predicted R-squared values were in good agreement with the adjusted R-squared values because the difference between them was less than 0.2. As illustrated in Table 7, all models had enough precision values of more than 4, signifying that the models could be used to navigate the design space. The final models’ equations for the responses of the mortar with all the model terms are presented in Equations (6)–(8). Using the analysis of variance model equations, the compressive strength, flexural strength, and water absorption of the cement-based mortar can be estimated.

Compressive strength = −5.8707 + 1.15764A + 54.77976B − 0.9AB − 0.0333A^2^ − 41.95536B^2^ (6)

Flexural strength = +0.070 + 0.29550A + 11.14167B + 0.035AB − 0.015150A^2^ − 10.375B^2^ (7)

Water absorption = +11.50527 + 0.15613A − 2.68333B(8)

**Figure 9:**
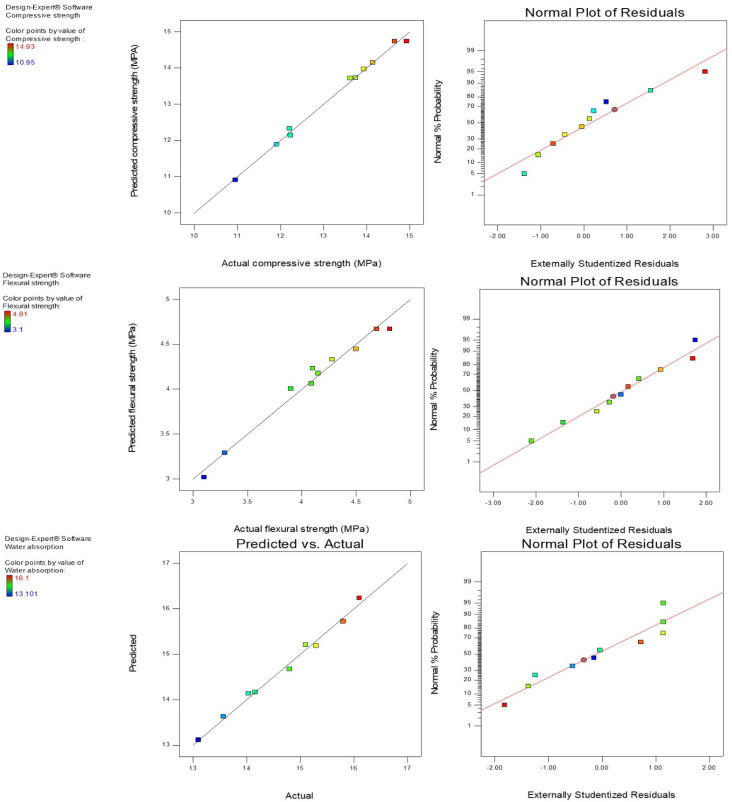
Predicted vs. actual and normal plot of residuals for output variables.

Plots of predicted vs. actual results of compressive strength, flexural strength, and water absorption graphically evaluated the competency and fitness of the response variables. In Figure 9, the predicted vs. actual results plot for the output variables showed that the models of the predicted responses were precise and accurate. The points were smoothly fitted along a straight line, showing a good relationship in the developed models between the experimental and predicted results. Hence, the models of the output variables were relevant and appropriate in estimating the compressive strength, flexural strength, and water absorption of the mortar. The normal probability plot is a graphical presentation used to determine the distribution of data and its sufficiency. The points were distributed along the straight equality line for all the dependent variables and, thus, showed that the data were normally distributed for all residual responses.

### 3.7. Optimization and validation

**Figure 10:**
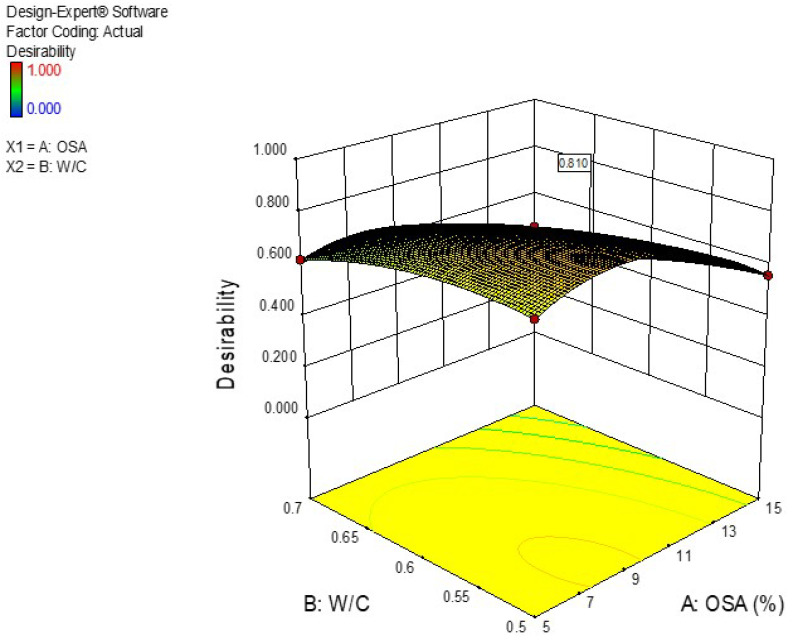
The desirable combination of OSA with the water-to-cement ratio.

**Figure 11:**
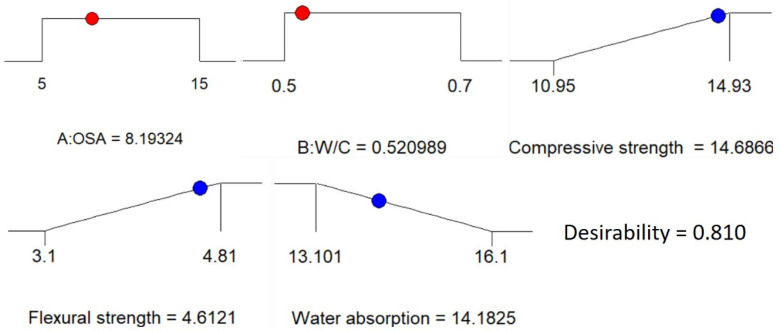
Optimization ramps of the geopolymer mortar.

Numerical multi-objective optimization was used to express the optimum content of OSA and the water-to-cement ratio to maximize the compressive strength and flexural strength and to minimize the water absorption of the developed cement-based mortar. The optimization study focused on recognizing the accepted values of input variables to accomplish the optimization goals. The responses influenced by the multiple factors were enhanced using the response surface method, which clarified the important role for the targeted dependent variables to improve the responses variables. For the optimization goals, the values for optimization are presented in Table 8. The design expert software obtained the maximum desired mixture fractions through synthesizing 8.19324 wt.% of OSA with 0.521 as the ratio of water to cement. Improved responses with an associated desirability of 81% were achieved. The numerical optimization results for the developed models are shown in the 3-D plot in Figure 10. Besides, the input variables and independents variables are presented graphically by the optimization ramps in Figure 11.

To validate the appropriateness of the optimization effects, an additional set of investigations were carried out by applying the optimized mixture proportion and two more mixtures to check the optimized mix proportion. The error in the laboratory and predicted values was assessed using Equation (9). The predicted results, experimental outcomes, and percentage error are presented in Table 9. According to the calculated percentage errors, it can be concluded that the experimental and predicted results were in excellent agreement with one another due to the reasonably small percentage errors.

(9) $$Error\left(\%\right)=\frac{Experimental\;model-predicted\;model}{Experimental\;model}\times 100\%$$

## 4. Conclusions

This study focused on optimization using the response surface technique by partially replacing cement with oily sludge ash in mortar. The water-to-cement ratio was varied from 0.4 to 0.8 to produce mortar suitable for repair application. Based on the results of this study, the following conclusions were made:

- The OSA contained a crystalline structure and packs of angular grains. The OSA had the sum of pozzolanic oxides as 70.60%, along with CaO of 9.82%. OSA had hydrophobic groups, including methylene and methyl aromatic rings.
- The influence of OSA inclusion in the cement-based mortar mixtures and water-to-cement ratios improved the compressive strength and flexural strength significantly. In addition, the water absorption trend increased linearly with the increase in both the ratios of water to cement and the amount of OSA in the mortar matrices, which could affect the mortar durability.
- The models for compressive strength and flexural strength were quadratic, signifying a good relationship between the variables.
- The outcome of the RSM optimization study shows that the best strength could be achieved by combining 8.19 wt.% of OSA, and a w/c ratio of 0.52. The experimental results were correlated with the predicted results and the obtained experimental results were close to the validation results.

## Figures and Tables

**Table 1:** Factors in uncoded values for the coded values of different parameters.

Notation	Parameter	Unit	Coded Values				
			−α	−1	0	+1	+α
			Uncoded values				
A	OSA	%	0	5	10	15	20
B	W/C	-	0.4	0.5	0.6	0.7	0.8

**Table 2:** Mix formulations of mortar.

Std	Run	Factor 1	Factor 2
		A = OSA (%)	B = W/C
7	1	10	0.4
4	2	15	0.7
10	3	10	0.6
6	4	20	0.6
3	5	5	0.7
1	6	5	0.5
9	7	10	0.6
5	8	0	0.6
2	9	15	0.5
8	10	10	0.8

**Table 3:** Mix formulations of mortar.

Run	Factor 1	Factor 2	Cement	OSA	Sand	Water
	A = OSA (%)	B = W/C	kg/m^3^	kg/m^3^	kg/m^3^	kg/m^3^
1	10	0.4	470.46	52.27	1568.18	209.09
2	15	0.7	415.96	73.40	1468.09	342.55
3	10	0.6	450.00	50.00	1500.00	300.00
4	20	0.6	400.00	100.00	1500.00	300.00
5	5	0.7	464.89	24.47	1468.09	342.55
6	5	0.5	485.55	25.56	1533.33	255.56
7	10	0.6	450.00	50.00	1500.00	300.00
8	0	0.6	500.00	0.00	1500.00	300.00
9	15	0.5	434.44	76.67	1533.33	255.56
10	10	0.8	431.25	47.92	1437.5	383.33

**Table 4:** Primary characterization of oily sludge.

Characteristics	Value
Moisture content (%)	61.4%
Total solid residue, TSR (%)	35.8%
pH value	6.44
Total organic carbon (TOC)	2.2%
Petroleum Hydrocarbon (PHCs)	Polycyclic aromatic hydrocarbon (PAH): 0.62%
	Aliphatic hydrocarbon compound: 86.26%
	Other compounds: 13.12%
Total Petroleum Hydrocarbon (TPH)	Diesel range organic (DRO): 84.3%
	Gasoline range organic (GRO): 0.02%
	Oil range organic (ORO) and residual range organic (RRO): 15.68%

**Table 5:** Chemical composition of OPC and OSA.

	SiO_2_	CaO	Al_2_O_3_	ZnO	Fe_2_O_3_	SO_3_	MgO	K_2_O	P_2_O_5_
OPC	18.99	65.02	4.39	-	3.09	5.27	1.72	0.41	0.77
OSA	14.9	9.82	10.2	1.01	45.5	10.6	2.77	1.08	1.85

**Table 6:** Analysis of variance for response models.

Responses	Factors	SS	Df	MS	F-Value	*p*-Value	Remarks
Compressive strength (Mpa)	Model	15.56	5	3.11	155.03	0.0001	Significant
	A-OSA	0.72	1	0.72	35.88	0.0039	
	B-W/C	2.50	1	2.50	124.65	0.0004	
	AB	0.81	1	0.81	40.35	0.0031	
	A^2^	11.52	1	11.52	573.90	<0.0001	
	B^2^	2.92	1	2.92	145.48	0.0003	
	Lack of Fit	0.041	3	0.014	0.35	0.8107	Not significant
Flexural strength (MPa)	Model	2.68	5	0.54	34.21	0.0022	Significant
	A-OSA	0.055	1	0.055	3.49	0.1351	
	B-W/C	0.11	1	0.11	7.03	0.0568	
	AB	1.225 × 10^−3^	1	1.225 × 10^−3^	0.078	0.7936	
	A^2^	2.38	1	2.38	151.93	0.0002	
	B^2^	0.18	1	0.18	11.40	0.0279	
	Lack of Fit	0.055	3	0.018	2.57	0.4232	Not significant
Water absorption (%)	Model	8.18	2	4.09	302.12	<0.0001	Significant
	A-OSA	7.31	1	7.31	540.40	<0.0001	
	B-W/C	0.86	1	0.86	63.85	<0.0001	
	Lack of Fit	0.095	6	0.016	0.30	0.9094	Not significant

Where SS: the sum of squares; Df: the degree of freedom, P: probability; F: Fisher statistical value; MS: mean square.

**Table 7:** Validation properties of response models.

Response Variable	SD	Mean	R^2^	Adj. R^2^	Pred. R^2^	AP
Compressive strength (MPa)	0.14	13.23	0.9949	0.9884	0.9704	34.834
Flexural strength (MPa)	0.13	4.09	0.9771	0.9486	0.8411	17.018
Water absorption (%)	0.12	14.68	0.9885	0.9853	0.9745	49.008

Where SD: standard deviation, R^2^: correlation coefficient, Adj. R^2^: adjusted correlation coefficient, Pred. R^2^: predicted R^2^, AP: adequate precision.

**Table 8:** Optimization benchmarks.

Variables and Responses	Unit	Goals	Lower Limit	Upper Limit
OSA	%	In range	5	15
W/C ratio		In range	0.5	0.7
Compressive strength	MPa	Maximize	10.95	14.93
Flexural strength	MPa	Maximize	3.10	4.81
Water absorption	%	Minimize	13.10	14.93

**Table 9:** Model validation.

Responses	OSA (wt.%)	W/C Ratio	Predicted Outcomes	Experimental Outcomes	Error (%)
Compressive strength (MPa)	8.19	0.52	14.69	15.15	3.1
	5	0.50	13.75	14.31	4.05
	15	0.70	12.21	12.54	2.72
Flexural Strength (MPa)	8.19	0.52	4.61	6.90	2.03
	5	0.50	4.1	4.25	3.70
	15	0.70	3.91	4.02	2.79
Water absorption (%)	8.19	0.52	14.18	14.64	3.24
	5	0.50	13.57	14.22	4.80
	15	0.70	15.80	16.68	5.60

## Data Availability

The data presented in this study are available on request from the corresponding author.
